# Clinical characteristics of children with omicron SARS-CoV-2 infection in Changchun, China from march to april 2022: A retrospective study

**DOI:** 10.3389/fped.2022.990944

**Published:** 2022-11-15

**Authors:** Yan-chun Li, Zhen Ma, Hua-ying Zhong, Hai-long You

**Affiliations:** Department of Pediatrics, The First Hospital of Jilin University, Changchun, China

**Keywords:** SARS-CoV-2, breakthrough infection, children, omicron variant, clinical characteristics 4 4

## Abstract

**Background:**

Recently, there was an outbreak in China of the Omicron (B.1.1.529) variant, the corresponding clinical characteristics of Chinese children with the Omicron variant of severe acute respiratory syndrome coronavirus 2 (SARS-CoV-2) infection were then reviewed and summarized retrospectively.

**Methods:**

From March to April 2022, a total of 134 children infected with the Omicron variant were included in the study. Data such as sex, age, clinical symptoms, laboratory examinations, and imaging features were collected for further analyses.

**Results:**

Half of the children were male and the median age was 5.67 years. The most SARS-CoV-2 Omicron variant was identified in mild (122, 91%), and the most three frequent symptoms were as cough (108, 80.6%), fever (75, 56%), and sore throat (38, 28.4%). Among age groups, no significant difference was observed in the distribution of symptoms, and no statistical difference was found in different clinical types among sex or age groups. Laboratory examinations revealed that white blood cells, neutrophils, and hemoglobin decreased; and monocytes, C-reactive protein (CRP), and aspartate aminotransferase (AST) increased. Further analyses showed that neutrophils, hemoglobin, CRP, and AST exhibited significant differences among age groups. Radiological abnormalities were found in nine cases, with small patchy high-density shadows. Of the 76 cured cases discharged from the hospital, the median hospital stay was 13 days (mean, 12 days).

**Conclusions:**

In China, most children with Omicron SARS-CoV-2 infection have mild presentation. The findings of this study may help other districts improve the management of children with Omicron SARS-CoV-2 infection in China.

## Introduction

On November 24, 2021, a novel severe acute respiratory syndrome coronavirus 2 (SARS-COV-2) variant, Omicron (B.1.1.529), was first identified in South Africa, which was responsible for a fourth wave of coronavirus disease 2019 (COVID-19) (1–3). In March 2022, an outbreak of Omicron variant infection was first recognized in Changchun, China.

The clinical manifestations caused by the Omicron variant strain are significantly different from those caused by the previous three viral strains and have garnered great attention (4, 5). Although many studies have revealed that the clinical characteristics caused by Omicron variant are significantly different from those caused by other variants (5–7), more investigation is needed in the Chinese population. Therefore, in this study, the clinical characteristics of Chinese children were collected and reviewed from March to April 2022, and then compared with the characteristics of children infected with Delta variants. Some novel findings were observed, which are inconsistent with those children infected with Omicron in South Africa (such as symptoms, oxygen use) (8) or with the Delta variant in this area. The findings of this study may improve the management of children with Omicron infection in other regions.

## Materials and methods

### Subjects

The retrospective study protocol was approved by the Ethics Committee of The First Affiliated Hospital of Jilin University (No. 2022-290). Written informed consent was obtained from the parents. From March 2022 to April 2022, children (≤4 years) admitted to the hospital with a diagnosis of COVID-19 were included for analysis. The diagnosis and treatment of COVID-19 were according to the guidelines issued by the National Health Commission of the People's Republic of China (9th version) (9). Diagnostic criteria were: previous contact history, symptoms such as fever and cough, radiological evidence; SARS-CoV-2 PCR (+), IgM or IgG (+, unvaccinated status). Discharge criteria were: recovery (temperature, respiratory symptoms, and radiological features) and sequential PCR (-, at least two times with a 1-day interval).

### Data collection

Data such as demographic characteristics, clinical type, clinical manifestations, treatment, laboratory examinations [e.g., SARS-COV-2 PCR, whole blood count, C-reactive protein (CRP), chemistry analysis], and radiological features were collected from the electronic medical records using a structured questionnaire.

### Statistical analyses

Statistical analyses was performed using SPSS (version 24.0). The quantitative data with normal distribution are expressed as the mean ± standard deviation (SD) and were compared using the *t*-test. Otherwise the median and interquartile range (IQR) were reported and compared using the Mann-Whitney test or Kruskal-Wallis test. The categorical data were reported with frequency (percentage) and compared using the chi square test and Fisher's exact test. *P* < 0.05 was considered statistically significant.

Statistical analysis was performed using R language (version 4.1.2). Continuous data that follow a normal distribution are expressed as the mean ± standard deviation, and otherwise are expressed as the median and interquartile range. Comparisons between groups were analyzed using the Mann-Whitney U test. Categorical data are expressed as frequencies (percentages), and comparisons were analyzed using the chi-square and Fisher's exact test. *P* < 0.05 is considered statistically significant.

## Results

### Baseline characteristics

A total of 134 children with COVID-19 were included in this study, of whom 67 (50.0%) were males. The median age was 5.67 (IQR, 2.58–9.92) years. The age distributions were as follows: neonates (<28 days; *n* = 1), infants (28 days to 1 year; *n* = 10), infants (1–3 years old; *n* = 31), preschool (3–6 years old; *n* = 26), school-age (6–12 years old; *n* = 43), and adolescence (12–14 years old; *n* = 23). There were 3 asymptomatic cases (2.2%), 122 mild cases (91%), 8 moderate cases (6%), and 1 severe case (0.7%). Table 1 shows the baseline characteristics of the children with COVID-19.

**Table 1:** Demographic and clinical types of children with omicron infection in Changchun, China.

Variables	Asymptomatic (*n*, %)	Mild (*n*, %)	Moderate (*n*, %)	Severe (*n*, %)	*P*
Sex					0.051
Female	1	64 (95.5)	1 (3.0)	1 (1.5)	
Male	2	58 (89.6)	7 (10.4)	0	
Age (Median, IQR)	2.50 (2.0,2.6)	5.58 (2.6,9.9)	8.75 (7.5,9.7)	7.25	
Neonates (<28 days)	0	1 (100)	0	0	0.253
Infants (28 days to 1 year)	0	10 (100)	0	0	
Infants (1–3 years)	3	28 (96.8)	0 (3.2)	0	
Preschool (3–6 years)	0	24 (92.3)	2 (7.7)	0	
School-age (6–12 years	0	37 (86.0)	5 (11.6)	1 (2.3)	
Adolescence (12–14 years)	0	22 (95.7)	1 (4.3)	0	

### Clinical types

In terms of clinical types, the median age was as follows: asymptomatic group, 2.50 (IQR, 2.0–2.6) years; mild group, 5.58 (IQR, 2.6–9.9) years, moderate group 8.75 (IQR, 7.5–9.7) years, and severe group, 7.25 years. There was no statistically significant difference in clinical classification between sex and age groups.

### Symptoms

The main symptoms of children infected with Omicron variant were cough (*n* = 108, 80.6%), fever (*n* = 75, 56%), sore throat (*n* = 38, 28.4%), muscle soreness (*n* = 8, 6%), diarrhea (*n* = 7, 5.2%), runny nose (*n* = 7, 5.2%), headache (*n* = 4, 3%), dyspnea (*n* = 3, 2.2%), fatigue (*n* = 1, 0.7%), and hypogeusia (*n* = 1, 0.7%). There was no statistically significant difference in the distribution of symptoms among age groups, which may be related to the small sample size of each symptom (Table 2).

**Table 2:** Main symptoms of included children with omicron infection.

Symptoms	Neonates	Infants	Infants	Preschool	School-age	Adolescence	$\chi^{2}/Z$	*P*
	<28 days	28 days to 1 year	1–3 years	3–6 years	6–12 years	12–14 years		
Fever	0	9	20	16	19	11	10.3	0.068
Fever duration (days)	—	2	2	2	2	2	2.13	0.712
Cough	1	8	25	20	35	19	0.545	0.990
Sore throat	0	0	5	11	13	9	10.5	0.062
Diarrhea	0	0	1	1	2	3	3.83	0.575
Muscle soreness	0	0	2	1	2	3	3.10	0.684
Runny nose	0	0	1	2	3	1	1.48	0.916
Fatigue	0	0	1	0	0	0	3.35	0.647
Anosmia	0	0	0	0	0	0	—	—
Hypogeusia	0	0	0	0	0	1	4.86	0.433
Conjunctivitis	0	0	0	0	0	0	—	—
Dyspnea	0	0	2	0	1	0	3.89	0.565
Headache	0	0	0	1	3	0	4.43	0.433

### Laboratory examinations

Routine blood examination showed that white blood cells (WBCs) were elevated in 8 (6.0%) patients and decreased in 27 (20.1%) patients; neutrophils were decreased in 51(38.1%) patients; lymphocytes were decreased in 4 (3.0%) patients; monocytes and its proportion were increased in 22 (16.4%) and 85 (63.4%) patients, respectively; and hemoglobin (HGB) was decreased in 7 (5.2%) patients. C-reactive protein (CRP), alanine aminotransferase (ALT), and aspartate aminotransferase (AST) were elevated in 58 (43.3%) patients, 3 (2.2%) patients, and 18 (13.4%) patients, respectively. Only two (1.5%) patients a had decreased level of albumin. Further statistical analyses showed that neutrophil, hemoglobin, CRP, and AST were significantly different among age groups (Tables 3, 4**)**.

**Table 3:** The distribution of abnormal lab examinations between age groups among children with omicron infection.

	Neonates (*n*, %)	Infants (*n*, %)	Infants (*n*, %)	Preschool (*n*, %)	School-age (*n*, %)	Adolescence (*n*, %)	Total	*P*
	<28 days	28 days to 1 year	1–3 years	3–6 years	6–12 years	12–14 years		
Number	1	10	31	26	43	23	8 (2.7)	0.047
White blood cell↑	0 (0.0)	3 (37.5)	3 (37.5)	1 (12.5)	1 (12.5)	0 (0.0)	8 (2.7)	0.047
White blood cell ↓	0 (0.0)	1 (3.7)	6 (22.2)	5 (18.5)	7 (25.9)	8 (29.6)	27 (9.2)	0.563
Neutrophils#↓	0 (0.0)	6 (10.2)	24 (40.7)	14 (23.7)	11 (18.6)	4 (6.8)	59 (20.1)	<0.001
Lymphocyte#↓	0 (0.0)	0 (0.0)	0 (0.0)	0 (0.0)	2 (50.0)	2 (50.0)	4 (1.4)	0.330
Monocytes%↑	1 (1.2)	5 (5.9)	20 (23.5)	17 (20.0)	24 (28.2)	18 (21.2)	85 (29.0)	0.455
Monocytes#↑	1 (4.5)	2 (9.1)	7 (31.8)	5 (22.7)	5 (22.7)	2 (9.1)	22 (7.5)	0.233
Hemoglobin↓	0 (0.0)	5 (71.4)	1 (14.3)	1 (14.3)	0 (0.0)	0 (0.0)	7 (2.4)	<0.001
Platenet↓	0 (0.0)	0 (0.0)	0 (0.0)	0 (0.0)	0 (0.0)	0 (0.0)	0 (0.0)	—
C reactive protein↑	0 (0.0)	0 (0.0)	7 (12.1)	14 (24.1)	22 (37.9)	15 (25.9)	58 (19.8)	<0.001
Alanine aminotransferase ↑	0 (0.0)	0 (0.0)	1 (33.3)	0 (0.0)	2 (66.7)	0 (0.0)	3 (1.0)	0.833
Aspartate aminotransferase ↑	0 (0.0)	7 (38.9)	11 (61.1)	0 (0.0)	0 (0.0)	0 (0.0)	18 (6.1)	<0.001
Albumin ↓	1 (50.0)	0 (0.0)	1 (50.0)	0 (0.0)	0 (0.0)	0 (0.0)	2 (0.7)	0.010
Creatine↑	0 (0.0)	0 (0.0)	0 (0.0)	0 (0.0)	0 (0.0)	0 (0.0)	0 (0.0)	—

**Table 4:** The level of lab examinations between age groups among children with omicron infection.

Variables	Neonates	Infants	Infants	Preschool	School-age	Adolescence	*H*	*P*
	<28 days	28 days to 1 year	1–3 years	3–6 years	6–12 years	12–14 years		
White bloo cell (10^9^/L)	9.4	7.5 (7.0,10.2)	5.8 (4.8,6.5)	5.7 (4.4,8.3)	5.11 (4.1,6.8)	4.3 (3.5,5.9)	15.11	0.010
Neutrophils# (10^9^/L)	1.3	0.6 (0.5,1.0)	0.9 (0.5,1.3)	1.5 (1.0,2.4)	2.0 (1.5,2.6)	2.2 (1.7,3.1)	38.07	<0.001
Neutrophils#	6.4	5.4 (4.6,7.0)	4.1 (3.1,5.2)	3.3 (2.4,4.0)	2.5 (2.0,3.3)	1.7 (1.5,2.2)	54.44	<0.001
Monocytes%	15.8	7.7 (6.7,8.8)	9.3 (6.6,12.6)	9.1 (7.5,10.9)	9.6 (7.2,11.7)	9.5 (8.2,12.9)	8.58	0.127
Monocytes#	1.5	0.5 (0.4,0.6)	0.5 (0.4,0.7)	0.5 (0.4,0.7)	0.5 (0.4,0.6)	0.5 (0.4,0.6)	3.91	0.562
C reactive protein (mg/L)	0.8	0.1 (0.1,0.6)	0.7 (0.3,0.9)	1.2 (0.2,3.5)	1.0 (0.3,3.5)	1.8 (0.9,5.2)	15.22	0.009
Alanine aminotransferase (U/L)	17	29.0 (24.0,36.0)	15.0 (13.0,20.0)	11.0 (10.0,12.8)	12.0 (9.0,15.5)	12.0 (9.0,16.5)	29.46	<0.001
Aspartate aminotransferase (U/L)	28.0	47.0 (46.0,55.0)	42.0 (32.5,47.0)	29.0 (27.0,32.0)	23.0 (21.0,30.5)	18.0 (15.5,19.5)	90.06	<0.001
Albumin (g/L)	34.7	38.5 (37.7,39.6)	41.4 (40.0,43.1)	42.2 (40.5,43.4)	41.4 (40.2,43.0)	42.1 (40.9,43.4)	14.39	0.013
Creatine (mmol/L)	13.0	14.0 (11.0,15.3)	20.0 (18.0,23.0)	26.0 (22.0,29.0)	33.0 (28.5,38.5)	46.0 (40.0,57.0)	91.70	<0.001

### Imaging

Nine cases had radiological abnormalities, and all patients showed small patchy high-density shadows including the right side (*n* = 8), left side (*n* = 1), right upper lobe (*n* = 6), right lower lobe (*n* = 2), and left lower lobe (*n* = 1). Most abnormalities recovered within 1 week, except two children (one was significantly improved after 3 days, and another showed that the primary lesion disappeared and a new lesion presented on the contralateral lung).

### Hospital stay

Most children were in good condition. Due to laryngeal obstruction, one child was treated with oxygen therapy, and the dyspnea was relieved after symptomatic treatment. No death was reported. To date, 76 patients were cured and discharged, with a median stay of 13 days (mean, 12 days).

### Vaccination status

The vaccination status was available in 131 (131/134, 97.8%) children. There were three children with incomplete data. Of the 131 children, 71 had not received the COVID-19 vaccine, 6 had their first vaccine dose, and 54 had their second vaccine dose. Statistical analysis showed no significant difference between the mild form and other types regarding age, vaccination status, and period between final vaccination and hospitalization (Table 5).

**Table 5:** Vaccination status among children with different types of COVID-19.

Variables	Total (*n* = 134)	Asymptomatic (*n* = 3)	Mild (*n* = 122)	Others (*n* = 9)	*P*
Sex, *n* (%)					0.193
Male	67 (50)	2 (66.7)	58 (47.5)	7 (77.8)	
Female	67 (50)	1 (33.3)	64 (52.5)	2 (22.2)	
Age, years (Median, IQR)	68 (31, 119)	30 (24, 31)	67 (31, 119)	104 (87, 115)	0.081
Vaccination status					**0**.**021**
No	71 (54.2)	3 (100)	67 (56.3)	1 (11.1)	
First-dose	6 (4.6)	0 (0)	6 (5)	0 (0)	
Second-dose	54 (41.2)	0 (0)	46 (38.7)	8 (88.9)	
Time period (*n* = 36)^a^	114.03 ± 46.26		113.62 ± 48.98^b^	117.25 ± 12.92^c^	0.741

^a^ Between final vaccination and hospitalization, with data of 36 children.

^b^ *n* = 32.

^c^ *n* = 4.

## Discussion

According to a report from Jilin CDC (http://wjw.jlcity.gov.cn/), the Omicron epidemic in Changchun is more severe than the previous COVID-19 epidemic. For example, the number of children with Omicron infection is larger than that caused by the Delta variant reported in other Chinese cities in 2021. Due to the zero COVID-19 strategy, little evidence is available characterizing children with Omicron infection in China. Hence, the characteristics of childhood COVID-19 were reviewed and summarized in the report.

Previously, very few COVID-19 children were studied. For example, in 2021, Huang et al*.* (4) studied 21 children infected with the Delta variant in southern China. Sheng et al*.* (10) reviewed 11 children with the Delta virus infection in central China. Moreover, during that year, a small cohort of children infected with Omicron was reported in the UK (*n* = 55) (11) and Spain (*n* = 15) (12). During an outbreak of Omicron variant in Shanghai, beginning March 7, 2022, a total of 376 children with Omicron was reviewed and reported in preprint without complete peer review (13). However, in 2021, large cohorts of childhood COVID-19 were reported. For example, in South Africa, a total of 6,287 children with confirmed COVID-19 cases were reported, including 869 cases (0–4 years), 1,231 cases (5–9 years), 2,023 cases (10–14 years), and 2,164 cases (15–19 years) (8). Similarly, a Omicron cohort of 22,772 children was reviewed in the USA (14). In general, limited evidence is available on childhood COVID-19 in China or abroad, especially for Omicron infection. Fortunately, in the March, 2022, a relatively large number (*n* = 134) of childhood COVID-19 cases were reported. Hence, a rapid review of their characteristics was performed, and we shared our experience with Chinese healthcare providers in improving the management of Chinese children with COVID-19.

First, the sex distribution was equal. Although previous study showed that sex could influence virus-driven T cell differentiation and maintenance in tissue sites and impact the anti-viral immune response (15), in this study, the male to female ratio was 1:1, and no statistical difference was found in the sex distribution of clinical types. Our findings were similar to previous Chinese reports in 2021. For example, the sex ratio of children infected with the Delta variant was 1:1.3 in Guangzhou (4) and 1:1.2 in Jingmen (10). In addition, similar findings were observed with the Omicron variant. For example, in the Qatar cohort, 54.6% of children were female (16). Other female proportion, such as 55.0% in Spain (12), 45.2% in Shanghai (13), and 47.3% in the USA (14) were also reported, displaying an equal distribution. However, this finding is not consistent with the COVID-19 in the adult. Previously, several reports point to sex differences in COVID-19 resulting from male patients having higher rates of infection, which is explained by social behaviour and human biology (17, 18). In addition, the median age of the children included was low and reported at 5.67 years old (IQR, 2.58–9.92), and approximately one-third of children were school-age, and more than half were 1–6 years old. This finding is inconsistent with previous reports. For example, the median age of children infected with Delta variant was 7 (IQR, 4–12) years (4). Jeane et al. (8) reported that children infected with the Omicron variant have a mean age of 4.2 years (SD 4.1) (8). In the USA COVID-19 cohort, the Omicron cohort was younger than the Delta cohort (14), and in most studies (12, 13, 16), the median age of Omicron children is reportedly between 6 and 7 years. In addition, our data also demonstrated that there was no significant difference in clinical types among age groups. In general, unlike other respiratory viral infections (such as respiratory syncytial virus) (19), the SARS-COV-2 infection in children has no age-dependent distribution. Interestingly, a previous study demonstrated that children aged 6 through 15 years had a longer persistence of viral genome in nasopharyngeal samples (20).

Second, most children infected with Omicron variants have mild symptoms. This means that during the epidemic in Changchun, the development of children's clinical type is different from previous epidemics, which happened in other Chinese cities. In the study, mild patients accounted for approximately 90% of all children. Nevertheless, as reported previously, children infected with Delta variant have relatively severe presentation, and the moderate type is significantly higher in previous studies (33.3% and 63.6%) than that of our study (4, 10). However, mild infection was significantly higher in children with Omicron infection than in those with the Delta variant (52.4% vs. 18.2%) (4, 10). Similar findings were observed in adulthood COVID-19 caused by the Omicron variant, which is that most patients have mild presentation (21, 22). In Shanghai, approximately one-third of Omicron-infected children were asymptomatic, and no severe disease was diagnosed (13). Wang et al. found that most children (82.9%) infected with Omicron variant had mild symptoms, mainly respiratory infection (23), which is consistent with the study by Ma et al. (24). According to the cohort in Qatar, among Omicron-infected children, 97.8% had mild, 2.2% had moderate, and none had severe/critical disease, Omicron variant infection (vs. Delta) was associated with significantly lower odds of moderate or severe/critical disease (16). Moreover, fewer comorbidities were reported in the Omicron cohort than in the Delta cohort (14).

In this study, only nine children had abnormal radiological features, with associated clinical characteristics (e.g., small patchy, high-density, unilateral). Similar radiological findings were reported by Huang et al. (4). In addition, radiological evidence also supported the mild presentation of children infected with the Omicron variant. No death was reported in the study, and only one child required oxygen therapy. Similarly, in the Spain cohort, only 2 of 94 (2.1%) patients were hospitalized, and no patient needed intensive care unit admission or died (12). Indirectly, this point is supported by that severe clinical outcomes in children infected with Omicron variant were significantly lower than those in the matched Delta cohort (14). However, in the UK cohort, 6.7% (3/45) had an oxygen requirement, 4.4% (2/45) required ventilation (1 invasive and 1 non-invasive), and 51.1% (23/45) received medication (11). In the UK cohort, more intervention were given, which may be explained by the cohort being younger. The data aremalso inconsistent with data from South Africa. According to the report from Jeané et al*.* (8), 20% of children required oxygen therapy, 5% were ventilated, and 3% were died during the study period. The difference in outcomes strengthens the role of geographical differences. In addition, the median hospital stay in our study was 13 days, which is shorter than that (median, 19 days) of the previous report by Huang et al. (4). In the UK cohort, the length of stay was shorter, ranging from 0 to 9 days with an average of 2 days (11). In a word, these characteristics such as clinical type, radiological evidence, treatment, and hospital period demonstrated that children with Omicron infection show a mild presentation.

Third, in the study, cough was the first symptom of children with Omicron infection, which accounted for 80.6% of all cases. Similar data were observed in the cohort conducted in UK, where fever and/or respiratory symptoms (86%) were the most common symptoms (11). However, another two reports in China (2021) showed that fever was the prominent symptom among children infected with Delta variant, with a proportion as high as 76.2% and 73% (4, 10). Moreover, 33.3% and 55% of children had cough in two studies (4, 10). Jeane et al. (8) reported that fever (61%) and cough (57%) were the most common symptoms among children with Omicron (B.1.1.529) variant infection, which is also inconsistent with our data. These results indicate that the symptoms caused by the Omicron variant are different from those caused by Delta variant and have geographical differences, due to the ethnic differences.

Fourth, laboratory examinations partly showed an abnormal status. For example, although the mean level of WBC (5.9 ± 2.2 × 10^9^/L), neutrophils (1.9 ± 1.5 × 10^9^/L), and lymphocytes (3.3 ± 1.7) were all within the normal range, a significant proportion of children showed an decreasing levels of WBCs (20.1%) and neutrophils (38.1%) and an increasing level of monocytes (63.4%) when compared with corresponding references. Increased percentage of monocytes was also observed in other studies (25). Although the underlying mechanism remains unclear and require further investigation, this may be explained by that (1) the SARS-CoV-2 infection could decrease the subset of neutrophils (26, 27), and increase the total of monocytes in mild group and decrease it in severe group (28, 29); (2) during the early stage, the decreased neutrophils and monocytes are known as an ongoing inflammatory status and risk factors of poor outcome among COVID-19 patients (29–31). Hence, patients may benefit from the normal or high numbers of neutrophils and monocytes. In addition, our study demonstrated that 43.3% of children infected with the Omicron variant showed elevated CRP. However, in a previous study of children infected with Delta variant, none (*n* = 21) showed elevated CRP (4), suggesting a different characteristic of children infected with the Omicron variant. Although our study had several interesting findings, the small sample size remains a concern, which may have led to significant selection bias. Therefore, further analysis of Chinese children with Omicron infection should be performed to improve the knowledge of the infection among Chinese children.

Fifth, vaccination status was also investigated. However, no significant difference was found between the mild form and other types regarding age, vaccination status, and period between final vaccination and hospitalization. In the study, half of the children had not received the SARS-CoV-2 vaccine. Recently, several studies have investigated the effects of COVID-19 vaccination against the Omicron variant. These findings support the efficacy of the SARS-CoV-2 vaccine. However, limitations remain for current vaccines. For example, the inactivated COVID-19 vaccine has a protective role for children, and more doses will be helpful to produce the IgG antibodies (23, 32). But, the production of IgG declines over time (32). In general, children are susceptible to infection with the Omicron variant (32). Meanwhile, children are more likely to be susceptible to vaccine breakthrough infections or reinfections due to the Omicron variant than previous variants (33).

## Conclusions

In conclusion, our study showed that in Chinese children with Omicron infection, cough and fever are the most common symptoms, their presentations are usually mild, no special treatment is required, and the outcomes are usually good.

## Data Availability

The original contributions presented in the study are included in the article/Supplementary Material, further inquiries can be directed to the corresponding author/s.
